# Mother’s Own Milk Provision During the First 12 Weeks of Life by Gestational Age

**DOI:** 10.1001/jamanetworkopen.2025.0024

**Published:** 2025-03-05

**Authors:** Aloka L. Patel, Joshua Wilson, Melissa Holmes, Tricia J. Johnson

**Affiliations:** 1 Department of Pediatrics, Rush University Children’s Hospital, Chicago, Illinois; 2 Rush University Medical School, Chicago, Illinois; 3 Department of Health Systems Management, Rush University, Chicago, Illinois

## Abstract

**Importance:**

Although mother’s own milk (MOM) is associated with reduced risk of neonatal complications of prematurity and improved neurodevelopmental outcomes, to our knowledge, there have been no large US studies reporting rates of MOM feeding initiation and sustained MOM feedings by gestational age (GA).

**Objective:**

To compare rates of MOM feeding initiation and continuation at 12 weeks for infants by GA.

**Design, Setting, and Participants:**

This retrospective cross-sectional study used state-level surveillance data for maternal characteristics and behaviors before and after pregnancy. Participants were women who gave birth from January 1 through December 31, 2021, in 36 US jurisdictions (33 states, the District of Columbia, New York City, and Puerto Rico) and completed the Pregnancy Risk Assessment Monitoring System (PRAMS) questionnaire. Data were analyzed from July 2023 to April 2024.

**Exposure:**

Infant GA, including early preterm (EPT; ≤27 weeks’ gestation), moderately preterm (MPT; 28-33 weeks’ gestation), late preterm (LPT; 34-36 weeks’ gestation), and early term to postterm (≥37 weeks’ gestation).

**Main Outcomes and Measures:**

MOM feeding initiation and continuation at 12 weeks after delivery. Weighted multivariable binary logistic regression models were used to test the association between MOM feeding initiation and continued provision at 12 weeks after delivery by infant GA.

**Results:**

Among 29 098 births, there were 0.4% EPT, 1.8% MPT, 6.7% LPT, and 91.1% early-term to postterm infants. Unadjusted MOM feeding initiation was highest for EPT infants (89.7% [95% CI, 85.0%-94.7%]) and lowest for LPT infants (81.8% [95% CI, 79.5%-84.1%]). For infants that initially received MOM, 71.6% (95% CI, 70.7%-72.6%) of early-term to postterm infants still received MOM at 12 weeks followed by 63.1% (95% CI, 52.9%-73.2%) of EPT infants, 61.2% (95% CI, 58.0%-64.3%) of LPT infants, and 58.6% (95% CI, 53.4%-63.8%) of MPT infants. After adjusting for maternal characteristics, LPT infants were 4.4 (95% CI, −6.7 to −2.1) percentage points less likely to initiate MOM feedings and those who received any MOM were 6.7 (95% CI, −9.9 to −3.5) percentage points less likely to receive MOM at 12 weeks compared with early-term to postterm infants. There were no differences in adjusted initiation or continuation rates among EPT (3.1 [95% CI, −1.4 to 7.5] percentage points for initiation and −0.0 [95% CI, −8.6 to 8.6] percentage points for continuation) or MPT (2.4 [95% CI, −0.5 to 5.3] percentage points for initiation and −3.3 [95% CI, −8.0 to 1.5] percentage points for continuation) infants compared with early-term to postterm infants.

**Conclusions and Relevance:**

This cross-sectional study found that MOM feeding initiation and continuation rates at 12 weeks after birth for LPT infants were substantially lower than rates for infants with other GAs. Research is needed to pinpoint barriers to MOM feeding initiation and continuation in this vulnerable population of infants.

## Key Points

**Question:**

Does the initiation and duration of mother’s own milk feeding vary by gestational age at birth?

**Findings:**

In this cross-sectional study using Pregnancy Risk Assessment Monitoring System data for 29 098 births in 36 US jurisdictions, late preterm infants were significantly less likely to have mother’s own milk feeding initiated and continued through the first 12 weeks of life compared with early and moderately preterm and early-term to postterm infants.

**Meaning:**

The findings suggest that research is needed to better understand barriers and develop strategies to increase mother’s own milk feeding rates for late preterm infants.

## Teaser

This cross-sectional study uses data from the Pregnancy Risk Assessment Monitoring System to compare the rates of initiating and continuing feeding with mother’s own milk at 12 weeks after birth among infants with different gestational ages.

## Introduction

Approximately 384 000 preterm infants are born annually in the US, representing 10.4% of total births.^1,2,3^ It is well established that breast milk from the mother—mother’s own milk (MOM)—protects all infants, including those born preterm, against infectious and chronic diseases later in life.^4,5,6^ National and international organizations recommend exclusive MOM feeding through 6 months of age and continued MOM feeding through 2 years of age.^4,7,8^ Healthy People 2030 proposes a US national goal of 42% of infants receiving exclusive breast milk at 6 months by 2030^9^; however, this goal is driven by a baseline rate that is predominantly based on full-term infants, and preterm infants are frequently subsumed within all births or excluded from analyses.

Despite the critical importance of MOM feeding for reducing risks of neonatal complications and later neurodevelopmental impairment in preterm infants,^4,10,11,12,13,14,15,16^ to our knowledge, there are no US national population-based statistics that report MOM feeding duration by gestational age (GA) at birth. The available data for preterm infants are frequently limited to infants in the neonatal intensive care unit (NICU)^17,18,19,20,21,22^ or to specific geographic areas^23,24^ and, therefore, may not be representative of all preterm births. Data are even more scarce within categories of preterm births^21,24,25,26,27,28,29,30,31^ despite late preterm (LPT; 34-36 weeks’ gestation), moderately preterm (MPT; 28-33 weeks’ gestation), and extremely preterm (EPT; ≤27 weeks’ gestation) births posing distinct challenges for mothers. For example, mothers of preterm infants are more likely to be pump dependent and to be ill at the time of delivery,^32^ requiring additional supports to facilitate successful initiation and sustained MOM feedings, compared with mothers of full-term infants. Mothers of MPT and LPT infants may be overlooked for lactation challenges, as their healthier and larger infants often have short NICU stays and may physically appear to be similar to full-term infants despite their greater risks for inadequate oral suction pressures and poor feeding at the breast compared with full-term infants.^33,34,35,36,37^

Data on the rates of MOM feeding initiation and continued MOM feedings are needed so that clinicians, researchers, and policy makers can quantify the gap between current MOM feeding rates and national goals and measure the effectiveness of initiatives seeking to increase initiation and duration of MOM feedings. Additionally, data by race and ethnicity are needed by GA to understand whether disparities observed in the overall population of infants are mirrored in preterm infants. Our objectives were to (1) evaluate whether differences in MOM feeding initiation and MOM feeding continuation at 12 weeks (ie, provision of MOM at 12 weeks for mothers who initiated MOM feeding) after birth exist by GA (EPT, MPT, LPT, and early term to postterm [≥37 weeks’ gestation]) and (2) investigate the extent to which differences in MOM provision at 12 weeks after birth are explained by MOM feeding initiation differences. As an exploratory aim, we examined MOM feeding initiation and continuation by key maternal sociodemographic variables.

## Methods

This was a retrospective cross-sectional study using Pregnancy Risk Assessment Monitoring System (PRAMS) data from January 1 through December 31, 2021. PRAMS is a surveillance system established by the Centers for Disease Control and Prevention to collect population-level data about maternal characteristics; health behaviors; health indicators before, during, and after pregnancy; and infant health indicators.^38^ Monthly samples are drawn from state birth certificate files. Women are surveyed by mail or telephone between 2 and 6 months after delivery, and completed surveys are linked to birth certificate data. Data used in this study included birth certificates and survey information from 36 jurisdictions, including 33 states, the District of Columbia, New York City, and Puerto Rico. These 36 jurisdictions included all states that had Automated Research File data available for release by the Centers for Disease Control and Prevention at the time of our data request in June 2023. The analysis was limited to women who completed the survey at least 12 weeks after delivery. Records were excluded if the infant died or records were missing GA, breastfeeding duration, or information for any maternal characteristic. This cross-sectional study followed the Strengthening the Reporting of Observational Studies in Epidemiology (STROBE) reporting guideline for observational studies. This study was reviewed by the Rush University institutional review board and was determined to not meet the definition for human participants research. Thus, the requirement for obtaining informed consent was waived.

Outcomes included MOM feeding initiation, defined as providing any maternal breast milk, and MOM feeding continuation, defined as MOM provision at 12 weeks after delivery, conditional on MOM feeding initiation. A secondary outcome was MOM provision at 12 weeks after delivery, not conditional on MOM feeding initiation. MOM feeding duration was determined based on the survey question, “How many weeks or months did you breastfeed?”

Infant GA at birth was classified as EPT, MPT, LPT, and early term to postterm. Maternal characteristics included age (<20, 20-24, 25-34, and ≥35 years), educational level (less than high school, completed high school, some college or associate’s degree, or bachelor’s degree or greater), marital status (married, other marital status), health insurance enrollment 1 month before pregnancy (Medicaid, private insurance, other insurance, or no insurance), use of the Special Supplemental Nutrition Program for Women, Infants, and Children (WIC) during pregnancy, type of delivery (vaginal or cesarean), number of previous live births (0, 1, 2, 3, or >3), birth of multiples (single, multiple), prenatal care (first trimester, second or third trimester, or no prenatal care), maternal body mass index (BMI; underweight, <18.5; normal, 18.5-24.9; overweight, 25.0-29.9; obesity, ≥30.0 [calculated as weight in kilograms divided by height in meters squared]), cigarette use in the first trimester of pregnancy, and race and ethnicity (Hispanic, non-Hispanic Black [hereafter, Black], non-Hispanic White [hereafter, White], and other). Other race and ethnicity included Alaska Native, American Indian, Chinese, Filipino, Japanese, Native Hawaiian, other Asian, and other as coded in the PRAMS dataset. PRAMS collected race and ethnicity from birth certificate records, which are self-reported by the mother and father.

### Statistical Analysis

Sampling for PRAMS occurs at the jurisdiction level, representing states and other jurisdictions (the District of Columbia, New York City, and Puerto Rico). Participating sites randomly sample new mothers every month based on birth certificates. We calculated the weighted probability, with their 95% CIs, of MOM feeding initiation and continuation at 12 weeks, with weights reflecting the underlying populations of the 36 PRAMS jurisdictions included in our analysis. Weighted binary logistic regression models were used to test the association of infant GA category with MOM feeding initiation and with MOM feeding continuation at 12 weeks. Models were adjusted for maternal characteristics, including maternal age, race and ethnicity, educational level, marital status, health insurance status in the month before pregnancy, WIC use during pregnancy, type of delivery, number of prior live births, birth of multiples, prenatal care, BMI, and cigarette use in the first trimester. The adjusted probability of MOM feeding initiation, MOM feeding continuation at 12 weeks conditional on MOM feeding initiation, and unconditional probability of MOM provision at 12 weeks were calculated by GA using predictive margins. Predictive margins calculate the adjusted probability of an outcome that isolates the effect of a specific factor (eg, GA) and accounts for covariate distribution without making assumptions on the distributions of these variables.^39,40^ In brief, we first computed the adjusted probability of the outcome (eg, MOM feeding initiation) for all infants in the sample assuming that they were EPT, holding the values of all other characteristics at their observed values. We then computed the adjusted probabilities of the outcome (eg, MOM feeding initiation) for infants assuming that they were MPT, LPT, or early term to postterm, holding the values of all other characteristics at their observed values. Using this method, each observation has an adjusted probability of the outcome for the 4 GA categories (EPT, MPT, LPT, and early term to postterm). By comparing the adjusted probabilities across the different GA categories, we evaluated the marginal effect estimates (ie, how the probability of the outcome changes as GA changes), holding all other characteristics constant at their observed values. All analyses were conducted in SAS, version 9.4 (SAS Institute Inc) and Stata, version 17 (StataCorp LLC) from July 2023 to April 2024. Two-sided *P* < .05 was considered statistically significant.

## Results

In total, 33 227 women (91.1% of the sample) completed the survey at least 12 weeks after delivery. Records were excluded from the analysis if the infant died (n = 319) or the records were missing GA (n = 32), breastfeeding duration (n = 1315), or any maternal characteristic (n = 2463). The 29 098 births in the PRAMS sample represented 1 507 321 births in 2021, or 41% of total births in the US.^1^ A total of 14.3% of mothers were Black; 18.1%, Hispanic; 58.0%, White; and 9.6% other race and ethnicity. Overall, 0.4% of infants were born EPT; 1.8%, MPT; 6.7%, LPT; and 91.1%, early term to postterm (Table 1). All maternal characteristics differed by infant GA except for WIC use during pregnancy and prenatal care in the first trimester. Mothers of early-term to postterm infants were more likely to be White, to be married, to have completed college, to have a singleton, and to have delivered vaginally and were less likely to have Medicaid insurance compared with mothers of preterm infants.

**Table 1.** Description of the Study Sample

Variable	Total births, unweighted No.	Births, weighted % (95% CI)				*P* value
		EPT	MPT	LPT	Early term to postterm^a^	
**Total values**						
Unweighted No. (%)	29 098	275 (0.9)	1435 (4.9)	3330 (11.4)	24 058 (82.7)	NA
Weighted No. (%)	1 507 321	5843 (0.4)	27 831 (1.8)	100 998 (6.7)	1 372 648 (91.1)	NA
**Characteristics**						
Maternal age, y						
<20	1211	3.9 (1.4-6.4)	4.5 (2.9-6.1)	4.4 (3.2-5.5)	3.7 (3.3-4.1)	.004
20-24	4996	16.3 (10.4-22.2)	15.8 (12.5-19.2)	16.9 (14.6-19.1)	16.9 (16.1-17.6)	
25-34	16 964	58.0 (48.3-67.8)	56.7 (52.0-61.3)	54.9 (51.7-57.6)	59.7 (58.7-60.6)	
≥35	5927	21.7 (12.3-31.2)	23.0 (19.3-26.6)	24.1 (21.5-26.8)	19.8 (19.0- 20.5)	
Maternal race and ethnicity						
Hispanic	6171	13.7 (8.4-19.1)	17.8 (14.8-20.9)	18.9 (16.4-21.3)	18.1 (17.4-18.8)	<.001
Non-Hispanic Black	4668	35.1 (26.3-43.8)	24.8 (20.8-28.9)	20.1 (17.9-22.3)	13.6 (12.9-14.2)	
Non-Hispanic White	13 000	38.5 (28.5-48.5)	48.3 (43.5-53.2)	53.5 (50.6-56.3)	58.6 (57.8-59.4)	
Other^b^	5259	12.7 (4.9-20.5)	9.0 (6.5-11.5)	7.6 (6.3-8.9)	9.7 (9.2-10.2)	
Educational level^c^						
<High school	3083	7.9 (4.4-11.4)	11.8 (9.4-14.2)	11.0 (9.2-12.8)	9.8 (9.2-10.4)	<.001
Completed high school	6912	34.6 (25.5-43.6)	32.7 (27.7-37.7)	25.9 (23.3-28.6)	24.1 (23.2-24.9)	
Some college or associate’s degree	8052	27.9 (19.3-36.5)	27.9 (23.7-32.1)	29.3 (26.7-31.9)	25.1 (24.2-25.8)	
≥Bachelor’s degree	10 948	29.6 (19.6-39.5)	27.5 (23.5-31.5)	33.6 (30.8-36.5)	41.0 (40.1-41.9)	
Married	17 373	47.3 (37.4-57.3)	47.9 (43.1-52.6)	56.4 (53.4-59.3)	62.4 (61.5-63.4)	<.001
Medicaid	8780	37.1 (28.4-45.8)	35.3 (30.3-40.2)	31.9 (29.1-34.7)	26.4 (25.6-27.2)	<.001
Maternal WIC use in pregnancy	9394	31.4 (22.8-40.1)	31.8 (27.5-36.1)	29.6 (27.4-32.6)	28.5 (27.7-29.4)	.30
Vaginal delivery	18 643	26.7 (18.5-34.9)	33.2 (28.7-37.7)	53.1 (50.2-56.1)	68.9 (68.0-69.7)	<.001
Primiparous						
Yes	11 696	45.4 (35.7-55.1)	44.3 (39.5-49.1)	37.8 (34.9-40.7)	40.0 (39.0-40.9)	.02
No	13 843	54.6 (44.9-64.3)	55.7 (50.9-60.5)	62.2 (59.3-65.1)	60.0 (59.1-61.0)	
Singleton	28 168	88.7 (83.1-94.3)	81.9 (77.9-85.9)	89.8 (88.3-91.3)	99.3 (99.2-99.4)	<.001
Prenatal care in first trimester						
Yes	25 730	93.3 (89.7-96.9)	87.0 (83.9-90.2)	90.6 (89.0-92.1)	89.5 (88.9-90.1)	.10
No	3368	6.7 (3.1-10.3)	13.0 (9.8-16.1)	9.4 (7.9-11.0)	10.5 (9.9-11.1)	
Maternal BMI^d^						
Underweight	792	1.2 (0.0-2.5)	2.9 (1.5-4.4)	3.3 (2.1-4.5)	2.6 (2.3-2.9)	<.001
Normal	11 251	34.7 (25.0-44.3)	33.3 (28.8-37.7)	37.2 (34.3-40.1)	40.1 (39.2-41.0)	
Overweight	7610	23.7 (13.7(33.6)	25.4 (21.4-29.4)	24.5 (22.0-26.9)	26.4 (25.6-27.3)	
Obesity	9088	40.4 (30.9-49.9)	38.4 (33.4-43.3)	35.0 (32.3-37.8)	30.9 (29.9-31.8)	
Smoking in first trimester	1602	6.3 (1.0-11.6)	8.2 (5.2-11.3)	6.4 (4.9-7.9)	4.8 (4.3-5.2)	.004

Abbreviations: BMI, body mass index (calculated as weight in kilograms divided by height in meters squared); EPT, extremely preterm (≤27 weeks’ gestation); LPT, late preterm (34-36 weeks’ gestation); MPT, moderately preterm (28-33 weeks’ gestation); NA, not applicable; WIC, Special Supplemental Nutrition Program for Women, Infants, and Children.

^a^ Gestational age of 37 weeks or longer.

^b^ Alaska Native, American Indian, Chinese, Filipino, Japanese, Native Hawaiian, other Asian, and other as coded in the Pregnancy Risk Assessment Monitoring System dataset.

^c^ N = 28 994 (missing = 104).

^d^ N = 28 741 (missing = 357). Underweight was defined as less than 18.5; normal, 18.5 to 24.9; overweight, 25.0 to 29.9; and obesity, 30.0 or higher.

Unadjusted rates of MOM feeding initiation were similar for mothers of early-term to postterm (88.2% [95% CI, 87.5%-88.8%]), MPT (88.0% [95% CI, 85.1%-91.0%), and EPT (89.7% [95% CI, 85.0%-94.7%]) infants, with a lower rate of MOM feeding initiation observed for mothers of LPT infants (81.8% [95% CI, 79.5%-84.1%]) (Table 2). For mothers who initiated MOM feeding, 71.6% (95% CI, 70.7%-72.6%) of mothers of early-term to postterm infants continued to provided MOM at 12 weeks compared with 61.2% (95% CI, 58.0%-64.3%) of mothers of LPT infants, 58.6% (95% CI, 53.4%-63.8%) of mothers of MPT infants, and 63.1% (95% CI, 52.9%-73.2%) of mothers of EPT infants.

**Table 2.** Unadjusted MOM Feeding Initiation and Continuation at 12 Weeks of Age Conditional on MOM Feeding Initiation

Gestational age	MOM feeding initiation (n = 29 098)		MOM feeding continuation at 12 wk (n = 25 796)	
	Weighted % (95% CI)	*P* value	Weighted % (95% CI)	*P* value
EPT	89.7 (85.0-94.7)	<.001	63.1 (52.9-73.2)	<.001
MPT	88.0 (85.1-91.0)		58.6 (53.4-63.8)	
LPT	81.8 (79.5-84.1)		61.2 (58.0-64.3)	
Early term to postterm^a^	88.2 (87.5-88.8)		71.6 (70.7-72.6)	

Abbreviations: EPT, extremely preterm (≤27 weeks’ gestation); LPT, late preterm (34-36 weeks’ gestation); MOM, mother’s own milk; MPT, moderately preterm (28-33 weeks’ gestation).

^a^ Gestational age of 37 weeks or longer.

After adjusting for maternal characteristics compared with early-term to postterm mothers, there were no significant differences in MOM feeding initiation for mothers of MPT (2.4 [95% CI, −0.5 to 5.3] percentage points) or EPT (3.1 [95% CI, −1.4 to 7.5] percentage points) infants. Mothers of LPT infants were 4.4 (95% CI, −6.7 to −2.1) percentage points less likely to initiate MOM feeding and 6.7 (95% CI, −9.9 to −3.5) percentage points less likely to continue providing MOM at 12 weeks compared with mothers of early-term to postterm infants (Table 3 and Figure). There were no differences in adjusted continuation rates among EPT (−0.0 [95% CI, −8.6 to 8.6] percentage points) and MPT (−3.3 [95% CI, −8.0 to 1.5] percentage points) infants compared with early-term to postterm infants. Additionally, we found significant differences in MOM feeding initiation by maternal race and ethnicity, with Hispanic and White mothers and mothers of other racial groups more likely to initiate MOM feeding compared with Black mothers. However, for those who initiated MOM feeding, only Hispanic mothers had higher rates of MOM feeding continuation at 12 weeks compared with Black mothers (73.1% [95% CI, 71.3%-75.0%] vs 68.5% [95% CI, 66.1%-71.0%]), with no significant difference between rates among White mothers and mothers of other racial groups and rates among Black mothers (Table 3). We found no difference in MOM feeding initiation by WIC use during pregnancy; however, for mothers who initiated MOM feeding, those who did not use WIC had a higher rate of MOM feeding continuation at 12 weeks (72.4% [95% CI, 71.3%-73.5%] vs 66.6% [95% CI, 64.7%-68.4%]). Mothers with less than a high school diploma were least likely to initiate MOM feeding compared with mothers with higher levels of education (Table 3). The probability of MOM feeding continuation was significantly higher for mothers with a bachelor’s degree or more compared with mothers with less than a high school diploma (78.0% [95% CI, 76.6%-79.5%] vs 64.4% [95% CI, 60.9%-67.9%]).

**Table 3.** Adjusted Probabilities and Marginal Effect Estimates

Characteristic	Mean (95% CI)			
	MOM feeding initiation		MOM feeding continuation at 12 wk conditional on initiation	
	Adjusted probability	Marginal effect estimate^a^	Adjusted probability	Marginal effect estimate^a^
Overall	87.8 (87.7 to 87.9)	NA	67.8 (67.6 to 67.9)	NA
Gestational age				
EPT	91.2 (86.8 to 96.7)	3.1 (−1.4 to 7.5)	71.1 (62.6 to 79.7)	−0.0 (−8.6 to 8.6)
MPT	90.5 (87.7 to 93.4)	2.4 (−0.5 to 5.3)	67.9 (63.2 to 72.5)	−3.3 (−8.0 to 1.5)
LPT	83.7 (81.5 to 86.0)	−4.4 (−6.7 to −2.1)^b^	64.5 (61.4 to 67.5)	−6.7 (−9.9 to −3.5)^b^
Early term to postterm^c^	88.1 (87.5 to 88.8)	Reference	71.2 (70.3 to 72.0)	Reference
Maternal race and ethnicity				
Hispanic	93.9 (93.0 to 94.8)	10.5 (8.7 to 12.4)^b^	73.1 (71.3 to 75.0)	4.6 (1.6 to 7.6)^b^
Non-Hispanic Black	83.4 (81.7 to 85.0)	Reference	68.5 (66.1 to 71.0)	Reference
Non-Hispanic White	86.2 (85.2 to 87.2)	2.8 (0.8 to 4.8)^b^	70.4 (69.2 to 71.6)	1.9 (−0.9 to 4.7)
Other^d^	90.5 (88.8 to 92.3)	7.2 (4.8 to 9.5)^b^	69.6 (67.1 to 72.2)	1.1 (−2.4 to 4.6)
WIC during pregnancy				
Yes	85.8 (84.7 to 86.9)	Reference	66.6 (64.7 to 68.4)	Reference
No	89.2 (88.4 to 89.9)	3.4 (1.9 to 4.8)	72.4 (71.3 to 73.5)	5.8 (3.5 to 8.1)^b^
Maternal educational level				
<High school diploma	80.9 (78.4 to 83.4)	Reference	64.4 (60.9 to 67.9)	Reference
Completed high school	84.5 (83.1 to 85.9)	3.6 (1.0 to 6.2)^b^	64.4 (62.3 to 66.7)	0.0 (−3.7 to 3.9)
Some college or associate’s degree	89.9 (88.9 to 90.9)	9.0 (6.3 to 11.7)^b^	67.6 (65.9 to 69.3)	3.2 (−0.7 to 7.1)
≥Bachelor’s degree	92.0 (90.9 to 93.1)	11.1 (8.2 to 14.1)^b^	78.0 (76.6 to 79.5)	13.6 (9.6 to 17.7)^b^

Abbreviations: EPT, extremely preterm (≤27 weeks’ gestation); LPT, late preterm (34-36 weeks’ gestation); MOM, mother’s own milk; MPT, moderately preterm (28-33 weeks’ gestation); NA, not applicable; WIC, Special Supplemental Nutrition Program for Women, Infants, and Children.

^a^ Marginal effect estimates are the differences in the adjusted probabilities.

^b^ Significantly different from the reference group.

^c^ Gestational age of 37 weeks or longer.

^d^ Alaska Native, American Indian, Chinese, Filipino, Japanese, Native Hawaiian, other Asian, and other as coded in the Pregnancy Risk Assessment Monitoring System dataset.

**Figure.**
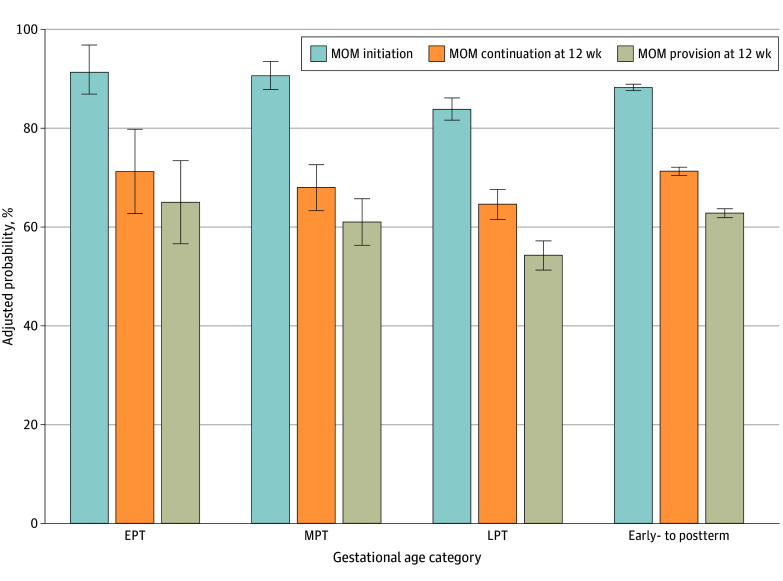
Adjusted Probabilities of Mother’s Own Milk (MOM) Feeding Initiation, MOM Feeding Continuation at 12 Weeks, and Overall MOM Provision at 12 Weeks MOM feeding continuation at 12 weeks includes only mothers who initiated MOM feeding; overall MOM provision at 12 weeks includes all mothers regardless of whether they initiated MOM feeding. Error bars represent 95% CI. EPT indicates extremely preterm; MPT, moderately preterm; LPT, late preterm.

In the supplemental analysis of overall MOM provision at 12 weeks (including mothers who did not initiate MOM feeding), 63.1% (95% CI, 62.2%-64.1%) of mothers of early-term to postterm infants, 50.0% (95% CI, 47.1%-53.0%) of mothers of LPT infants, 51.6% (95% CI, 46.8%-56.4%) of mothers of MPT infants, and 56.7% (95% CI, 47.1%-66.3%) of mothers of EPT infants provided MOM at 12 weeks (eTable 1 in Supplement 1). After adjusting for maternal characteristics, mothers of LPT infants were 8.5 (95% CI, −11.5 to −5.4) percentage points less likely to provide MOM at 12 weeks compared with mothers of early-term to postterm infants (eTable 2 in Supplement 1).

## Discussion

In this cross-sectional study of 2021 PRAMS data, LPT infants were consistently less likely to receive MOM feeding at birth and at 12 weeks compared with EPT or MPT and early-term to postterm infants. Over the past 2 decades, MOM feeding rates have increased, with 83% of infants ever receiving MOM in 2020 compared with 70% of infants in 2000.^41^ However, only 25% of infants were exclusively fed MOM through 6 months of age in 2020—below current targets.^4,7,8,9^ Yet, US national data on MOM feeding initiation or duration by subgroups of infants and mothers are limited,^21,42^ often including at-risk subgroups within the broader national data. Preterm infants have been considered particularly vulnerable for suboptimal MOM feedings due to the multiple barriers encountered by their mothers in provision of MOM,^25,43,44,45,46,47,48,49,50,51,52,53^ but the focus of many quality improvement efforts has been EPT and very preterm infants,^45,46,50,54,55^ largely ignoring MPT and LPT infants.

Our adjusted analyses of 2021 PRAMS data revealed that LPT infants were consistently less likely to receive MOM at birth and at 12 weeks compared with EPT or MPT and early-term to postterm infants. LPT infants account for the largest subgroup of all preterm infants and account for approximately 7.6% of all US live births.^3^ LPT infants are known to be at greater risk for neonatal morbidity, difficulty feeding, and readmission compared with their full-term counterparts.^27,34,35,36,56,57,58,59,60,61,62^ Specifically regarding direct breastfeeding, LPT infants are at particular risk due to their immature sucking ability, with weak suction patterns resulting in less robust milk transfer, which may in turn negatively impact milk supply. Their neurologic immaturity and limited ability to regulate sleep and wake cycles may result in parental perception of an LPT infant being satiated when the infant is falling asleep. These factors may result in inadequate nutrition, dehydration, and jaundice, with higher rates of readmission in the first 2 weeks after birth for LPT infants compared with full-term infants.^27,34,35,36,56,57,58,59,60,61,62,63,64,65^ Improving MOM feeding rates in this sizeable population of LPT infants may impact both short-term and long-term clinical outcomes, which would translate to future educational and economic benefits and lower medical costs.^66^

Global efforts to better understand and manage the breastfeeding challenges experienced by LPT infants are under way.^27,28,58,67,68,69,70,71,72,73,74^ In-hospital lactation and maternity care practices, such as mother-infant separation, type of feeding, and staff training, are associated with later MOM provision in LPT infants.^27,58,67,69^ Formula feeding as the first feeding is associated with lower exclusive MOM provision at 6 weeks.^27^ A Danish study demonstrated that training of NICU nurses in breastfeeding-supportive practices was associated with increased rates of exclusive breastfeeding at discharge from the NICU in preterm infants, the majority of whom were MPT or LPT.^74^ Site of infant care has been an inconsistent factor in previous observational studies.^58,67,73^ Colaizy and Morriss^58^ found a positive association of NICU admission with MOM provision, while Hannan et al^67^ found that LPT infants in the NICU were significantly less likely to be breastfed at 10 weeks (estimated probability, 0.86 [95% CI, 0.76-0.99]) despite no difference in initiation rates. These discrepant findings may be explained by variations in within-hospital lactation practices between the NICU and well-baby units. The Early Bloomers program in Boston focuses on an interdisciplinary breastfeeding program designed to support nursing education and consistency of care in the well-baby nursery specifically for LPT infants due to their physiologic immaturity.^69^ While clinical outcomes are not available yet, the focus on breastfeeding education for LPT infants is supported by our findings that LPT infants were the least likely to receive MOM. Postdischarge interventional studies have demonstrated mixed results. A randomized clinical trial^75^ of home lactation consultant visits for LPT and early-term infants found no effect of the intervention on exclusive breastfeeding in LPT infants, although that study was conducted in the context of early discharge with a relatively short follow-up period of 5 to 12 days post partum. By contrast, recent quasi-experimental studies demonstrated a positive association between in-home breastfeeding education and rate of any MOM feeding at 3 to 4 weeks post partum.^70,71,72^ Many quality improvement efforts focused on EPT and very preterm infants^45,46,50,54,55^ have indicated that quality improvement methods could be effective in increasing MOM provision for LPT infants. However, due to the relatively short length of hospitalization, quality improvement approaches for LPT infants would require both in-hospital interventions to improve initiation rates and outpatient interventions to provide or improve lactation support in the home after the infant is discharged from the hospital.

### Limitations

Limitations of this study include small sample sizes within the youngest GA categories, which prevented us from investigating the interaction of GA and race and ethnicity with MOM feeding duration. Disparities in MOM feedings based on race and ethnicity are well documented in the US for all infants regardless of GA.^20,21,76,77^ The underlying reasons for these disparities are likely multifactorial, including structural factors, such as availability of hospital lactation resources; economic factors, such as access to paid parental leave and need to return to work; social factors, such as lack of education regarding health benefits associated with MOM; and lack of breastfeeding role models due to historically low rates of breastfeeding in Black families.^76,77,78,79,80,81^ Strategies to address these structural factors have been proposed, instituted, or are undergoing evaluation.^28,46,54,55,82,83^ While the PRAMS data are rich in maternal characteristics, limited data were available regarding NICU hospitalization; duration of hospitalization; early in-hospital lactation practices, such as timing of first feeding or pumping and type of first feeding; and other maternal health conditions known to be negatively associated with lactation, such as diabetes^84,85^ Additionally, infant feeding data at 12 weeks did not include information about exclusive MOM feedings compared with combined MOM and formula feedings, further limiting our ability to assess current MOM rates relative to recommendations for exclusive MOM feedings. The data were self-reported and may be subject to recall bias. Another limitation is the time frame used in the survey, which was shorter than 6 months and thus did not provide data about a key outcome: exclusive MOM feeding at 6 months of age.

## Conclusions

In this cross-sectional study using 2021 PRAMS survey data, similar MOM feeding initiation and continuation rates were identified among EPT, MPT, and early-term to postterm infants. However, a difference existed in MOM feedings for LPT infants compared with other infants, with disparities identified in both MOM feeding initiation and MOM feeding duration for infants who received any MOM, which translated into differences in overall MOM provision at 12 weeks compared with infants with other GAs. Previous investigators^18,20,77^ have sought to determine barriers and facilitators to continued MOM provision in various subgroups of infants. The present data suggest the continued need for such investigations and novel strategies to assist families in meeting recommendations, particularly for families of LPT infants.

## Notes

Supplement 1. **eTable 1.** Unadjusted MOM Provision at 12 Weeks for All, Not Contingent on MOM Initiation, N = 29 098 **eTable 2.** Adjusted MOM Provision at 12 Weeks for All, Not Contingent on MOM Initiation, Adjusted Probabilities and Marginal Effects, N = 29 098

Supplement 2.
Data Sharing Statement
