# Supplementary material for: Mother’s Own Milk Provision During the First 12 Weeks of Life by Gestational Age
Source: JAMA Netw Open. 2025 Mar 5;8(3):e250024. doi: 10.1001/jamanetworkopen.2025.0024 (PMC11883506; doi:10.1001/jamanetworkopen.2025.0024)
Supplement: Supplement 1. — eTable 1. Unadjusted MOM Provision at 12 Weeks for All, Not Contingent on MOM Initiation, N = 29 098 eTable 2. Adjusted MOM Provision at 12 Weeks for All, Not Contingent on MOM Initiation, Adjusted Probabilities and Marginal Effects, N = 29 098 [file jamanetwopen-e250024-s001.pdf]

Supplementary Online Content

Patel AL, Wilson J, Holmes M, Johnson TJ. Mother's own milk provision during the first 12 weeks of life by gestational age. *JAMA Netw Open*. 2025;8(3):e250024. doi:10.1001/jamanetworkopen.2025.0024

eTable 1. Unadjusted MOM Provision at 12 Weeks for All, Not Contingent on MOM Initiation, N = 29,098

eTable 2. Adjusted MOM Provision at 12 Weeks for All, Not Contingent on MOM Initiation, Adjusted Probabilities and Marginal Effects, N = 29,098

This supplementary material has been provided by the authors to give readers additional information about their work.

© 2025 Patel AL et al. *JAMA Network Open*.

eTable 1. Unadjusted MOM Provision at 12 Weeks for All, Not Contingent on MOM Initiation, N = 29,098

Gestational age category	Weighted % (95% CI)	p-value
		<.001
EPT	56.7 (47.1, 66.3)	
MPT	51.6 (46.8, 56.4)	
LPT	50.0 (47.1, 53.0)	
Term	63.1 (62.2, 64.1)	

Notes: EPT: extremely preterm (≤27 weeks gestational age), MPT: moderately preterm (28-33 weeks gestational age), LPT: late preterm (34-36 weeks gestational age), Term (37 weeks gestational age or longer)

© 2025 Patel AL et al. *JAMA Network Open*.

eTable 2. Adjusted MOM Provision at 12 Weeks for All, Not Contingent on MOM Initiation, Adjusted Probabilities and Marginal Effects, N = 29,098

Characteristic	Adjusted Probability Mean (95% CI)	Marginal Effect Mean (95% CI)
**Overall**	60.7 (60.4, 60.9)	--
**Gestational Age**		
EPT	64.9 (56.5, 73.3)	2.2 (-6.2 to 10.6)
MPT	60.9 (56.2, 65.6)	-1.8 (-6.6 to 3.0)
LPT	54.2 (51.3, 57.2)	-8.5 (-11.5 to -5.4) [a]
Term	62.7 (61.8, 63.6)	REF
**Maternal Race and Ethnicity**[b]		
Black	57.4 (55.1, 59.8)	REF
Hispanic	68.6 (66.7, 70.4)	11.1 (8.2, 14.0) [a]
White	60.8 (59.6, 62.0)	3.5 (0.7, 6.1) [a]
Other Race	62.8 (60.2, 65.3)	5.3 (1.9, 8.8) [a]
**WIC during Pregnancy**		
Yes	57.0 (55.3, 58.8)	REF
No	64.4 (63.3, 65.4)	7.3 (5.1, 9.5) [a]
**Maternal Education**		
Less than high school diploma	52.1 (48.8, 55.4)	REF
High school diploma/GED	54.4 (52.3, 56.4)	2.3 (-1.3 to 5.8)
Some college or associate’s degree	60.6 (58.9, 62.2)	8.5 (4.8, 12.2) [a]
Bachelor’s degree or more	71.1 (69.5, 72.7)	19.0 (15.0, 23.0) [a]

[a] denotes value is significantly different from the reference group.
Notes: EPT: extremely preterm (≤27 weeks gestational age), MPT: moderately preterm (28-33 weeks gestational age), LPT: late preterm (34-36 weeks gestational age), Term (37 weeks gestational age or longer).
[b]Other race includes Alaska Native, American Indian, Chinese, Filipino, Japanese, Native Hawaiian, other Asian, and Other as coded in the PRAMS dataset.

© 2025 Patel AL et al. *JAMA Network Open*.
