# Supplementary material for: Mother’s Own Milk Provision During the First 12 Weeks of Life by Gestational Age
Source: JAMA Netw Open. 2025 Mar 5;8(3):e250024. doi: 10.1001/jamanetworkopen.2025.0024 (PMC11883506; doi:10.1001/jamanetworkopen.2025.0024)
Supplement: Supplement 2. — Data Sharing Statement [file jamanetwopen-e250024-s002.pdf]

Data Sharing Statement

Patel. Mother's Own Milk Provision During the First 12 Weeks of Life by Gestational Age. *JAMA Netw Open*. Published March 05, 2025. doi:10.1001/jamanetworkopen.2025.0024

Data
Data available: No

Additional Information
Explanation for why data not available: The data are publicly available from the CDC Pregnancy Risk Assessment Monitoring System.
